# Corrigendum/Erratum to “Global legislation regulating the donation, procurement and distribution processes of organs and tissues from deceased donors for transplants: A scoping review” [Heliyon 10 (4) (February 2024) e26313]

**DOI:** 10.1016/j.heliyon.2024.e31739

**Published:** 2024-05-22

**Authors:** Aline Moraes da Silva, Patrícia Trindade Benites, Maria Eduarda Gonçalves Zulin, Marcos Antonio Ferreira Júnior, Andr'eia Insabralde de Queiroz Cardoso, Elenir Rose Jardim Cury

**Affiliations:** a Doctoral Student in Health and Development of the Midwest Region at the Federal University of Mato Grosso do Sul, Campo Grande, MS, Brazil; b Universitary Hospital Maria Aparecida Pedrossian, Brazil; c Master Student in Nursing at the Federal University of Mato Grosso do Sul, Campo Grande, MS, Brazil; d Graduate and Post-Graduation Program in Nursing, Integrated Health Institute, Federal University of Mato Grosso do Sul, Campo Grande, MS, Brazil; e Post-Graduation Program in in Health and Development of the Midwest Region at the Federal University of Mato Grosso do Sul, Federal University of Mato Grosso do Sul, Campo Grande, MS, Brazil

In the original published version of this article, there was an issue with the interpretation of a citation; "Regarding legislation for obtaining organs and tissues from deceased donors for transplants, **the majority of countries (91.45 %), except Nicaragua [40], recognize BD as irreversible** and mandatory notification to organ procurement institutions and tissues.". The corresponding author has provided a corrected version to Table 2, the 4th paragraph results, reference no. 40 and a correction to a typo in table 2. The authors would also like to make additional changes to table 2. The correct version of these can be found below.

Table 2-

Fourth paragraph results -

Regarding legislation for obtaining organs and tissues from deceased donors for transplants, all of countries recognize BD as irreversible and mandatory notification to organ procurement institutions and tissues.

Reference 40-

[40] Diário Oficial la gazeta. Law 847, “Law of donation and transplantation of organs, tissues, and cells for human beings” [Internet]. 2013 p. 207. Available from: http://legislacion.asamblea.gob.ni/SILEG/Iniciativas.nsf/0/cf9bbf45111a551c06257a710083d00f/$FILE/LeyNo.847trasplantedeorganos.pdf

Correct a typo in table 2: regarding the country Argentina. It is currently opt-in, but it is opt-out.

Further corrections to table 2.

**Figure:**
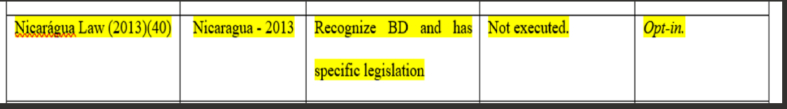

The authors/publisher apologize for the errors. Both the HTML and PDF versions of the article have been updated to correct the errors.

## Declaration of competing interest

None, no COIs.

